# Eye Troubles

**Published:** 1892-02

**Authors:** 


### EYE TROUBLES.

Inflammation of the conjunctiva or membrane which shields the front of the eyeball from the air and takes the rub of the eyelids, is indicated by the glued state of the eyes in the morning, and more especially by their bloodshot condition, the vessels being bright red in color, and winding about in great irregularity, with no discernible order or plan. Eyes are sometimes inflamed by being held too near the heat of a lamp, and relief may be obtained by shading the eyes with any old scrap of green paper, such as handbills are sometimes printed on. Weakness of the ciliary muscle, or an error of refraction, may be the cause of the evil. A refractive error might be corrected by proper spectacles ; and if the aching has increased under the use of the various glasses which have been tried, it points to a refractive error wrongly corrected as one cause of the trouble. Test each eye for astigmatism, and for long or short sight. Get properly suited with spectacles focussed for reading, writing and indoor work. And for the inflammation, wash the eyes with Goulard water ; also drop a few drops of the following lotion in the outer corner of each eye two or three times a day :—Hydrochlorate of cocaine, 8 grains ; boric acid, 3 drachms ; glycerine, 1 oz.; elder flower water, 6 oz.; and water to make 8 oz. Cold water should not be used for bathing the eyes when inflammation is present. Tepid water may be used night and morning, keeping the eye carefully closed the while.

